# An analysis of 3105 Medico Legal Cases at Tertiary Care Hospital, Rawalpindi

**DOI:** 10.12669/pjms.334.11696

**Published:** 2017

**Authors:** Romana Malik, Iffat Atif, Farah Rashid, Maqbool Abbas

**Affiliations:** 1 Dr. Romana Malik, DMJ. Forensic Medicine Department, Yusra Medical and Dental College (YMDC), Islamabad, Pakistan; 2 Dr. Iffat Atif, MPH. Community Medicine Department, Yusra Medical and Dental College (YMDC), Islamabad, Pakistan; 3 Prof. Dr. Farah Rashid, MPH, NLP, FRSPH. Community Medicine Department, Yusra Medical and Dental College (YMDC), Islamabad, Pakistan; 4 Dr. Maqbool Abbas, MBBS. Forensic Medicine Department, Yusra Medical and Dental College (YMDC), Islamabad, Pakistan

**Keywords:** Assault, Blunt trauma, Injuries, Medico legal cases, Road Traffic Accidents

## Abstract

**Background and Objectives:**

Medico legal cases are essential component of medical practice and comprise most important constituent of emergencies. The reporting of such cases is imperative to recognize theirsocioeconomic burden on any country. The present study was conducted to scrutinize different categories of medico legal cases and characteristics of the victims at casualty department oftertiary care hospital Rawalpindi. The objective of the study was to find out the frequency ofvarious categories of medico legal cases and major characteristics ofvictims at tertiary care hospital, Rawalpindi.

**Methods:**

This was a cross-sectional study on 3105 registered cases in medico legal record of the casualty department of Benazir Bhutto hospital, Rawalpindi from January 2015 to December 2015. The hospital is located on the main road in densely populated central area of the city. The data wascollected on age, sex, month-wise distribution of various medico legal cases, weapon inflicting the injury, blunt trauma or physical assault, firearm injuries and road traffic accidents. The data thus obtained was analyzed using SPSS; observations were presented in tables and graphs.

**Results:**

Out of all 3105 registered medico legal cases, reported cases caused by Road Traffic Accident 1230 (40%) followed by blunt injury or physical assault 966 (32%) cases, 19% by sharp weapons, 5% by poisoning, and 4% by firearm injuries. In our study out of 3105 cases, almost three quarter of victims (73%) were below 30 years of age, with a decreasing frequency beyond this age, males were predominantly inflicted 2516(81%) as compared to females 589 (19%). The reported road traffic accidents cases from urban areas were high (74%) as compared to those from rural locality (37%). In cases of blunt trauma, sharp weapon injuries and firearm injuries, there was a huge preponderance of victims from rural areas (65%), (62%) and 61% respectively, with urban cases constituting less.

**Conclusion:**

Road traffic injuries are one of the foremost causes of medico legal cases followed by blunt trauma and sharp weapon injuries. The emerging medico legal cases are neglected epidemic in most of the developing countries comprising a considerable public health problem.

## INTRODUCTION

Any case of injury or ailment where some criminality is involved is called a Medico Legal Case (MLC).^1^ A medico legal case is where a person is injured or harmed in any way and needs medical attention for it. The injury cases suggestive of criminal offense (blunt injuries and sharp edged weapons), burn injuries, vehicular accidents, suspected homicide (firearm injuries), poisoning, and sexual assault are classified as medico legal cases.^2^

Medico legal cases are essentialcomponent of medical practice and comprise most important constituent of emergencies.^3^ A medico legal case is a case of injury or illness where the attending doctor after obtaining history and examining the patient, considers that some investigation by law enforcement agencies is essential to establish and secure responsibility for the case.^4^ Medico legal cases form a major component of emergencies brought to casualty department of all teaching hospitals, which is mainstay to deal with all such cases.^5^ The provision of legal and medical services to such cases constitutes substantial proportion of workload in these hospitals.^5^,^6^

The reporting of medico-legal cases is imperative to recognize the burden of medico legal cases, calculate their risk and for the avoidance of preventable casualties in future.^6^ The idea is to initiate legal proceeding at the earliest so that maximum evidence can be collected to study the crime pattern in the area.^7^ The mortalities and morbidities from all medico legal causes has been increasing at an alarming rate in our countryand also throughout the world, yet to be controlled effectively; by the year 2020 mortality from injury will be more than those from communicable diseases.^8^ Despite this documentation, injuries are still not well recognizedas a major public health problem in ourcountry.^9^ The acquired data is mandatory to evaluatesocioeconomic consequences of the injuries and the development ofpreventive strategies in future.

The present study was conducted to find out different categories of medico legal cases and characteristics of the victims documentedat the casualty department ofone of the major public sector tertiary care hospital Rawalpindi. The objective was to determine various categories of medico legal cases and major characteristics of victims at tertiary care hospital, Rawalpindi. Determine

## METHODS

A cross sectional study was conducted onall the registered medico legal cases (3105)reported tocasualty department of public sector Benazir BhuttoHospital, Rawalpindi. The total duration of this study wasfrom 1^st^ January 2015 to 31^st^ December 2015. The cases included were of medico legal standpoint regardless of mode of injury i.e., accidental, homicidal and suicidal. The data was collected on age, sex, month-wise distribution of MLC, weapon inflicting the injury, blunt trauma or physical assault, firearm injuries androad traffic accidents from medico-legal registers. Ethical approval was taken from ethical review board of the institution and the administration of concerned hospital ensuring data confidentiality. The data thus obtained was analyzed using SPSS; observations were presented in tables and graphs.

## RESULTS

**Fig. 1:**
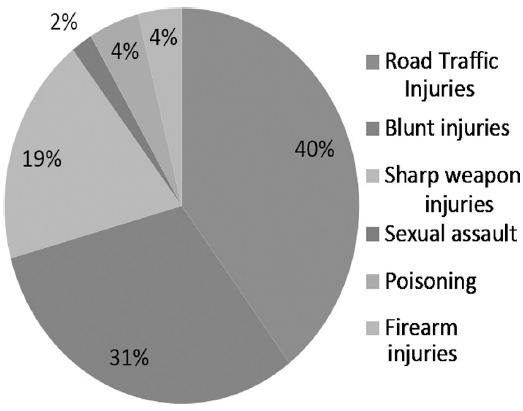
Categories of Medico legal cases documented at casualty department.

A total number of 3105 medico legal cases were included in the study from 1^st^ January 2015 to 31^st^ December 2015. The casualty department documented various types of medico legal cases shown in Fig. 1.

In our study out of 3105 cases, maximum number of cases 1283 (42%) were reported having predilection of age during third decade of life i.e., in the age group 21-30 years followed by age group 11-20 years which showed 955 (31%) cases. Almost three quarter of victims (73%) were below 30 years of age, with a decreasing frequency beyond this age (Table-I).

**Table I:** Types of medico legal cases in different age groups.

*Age groups*	*Road Traffic Accidents*	*Blunt injuries*	*Sharp weapons*	*Sexual Assault*	*Poisoning*	*Firearm injuries*
<20 years	21%	22%	38%	34%	11%	31%
21-30 years	52%	62%	51%	48%	32%	42%
31-40 years	18%	11%	8%	15%	24%	15%
> 40 years	9%	5%	3%	3%	33%	12%

In respect to gender distribution, males were predominantly inflicted 2516 (81%) as compared to females 589 (19%) with a male to female ratioof 4:1. In our study most of the medico legal cases were reported from urban areas 1879 (61%)) as compared to rural ones 1226 (39%). The reporting of sexual assault was least owing to our socio-cultural taboos and norms and can also be attributed to less reporting of medico legal cases amongst female victims with male to female ratio of 4:1.

The present study revealed maximum number of cases were registeredduring the month of August 314 (10%) followed by November 298 (9%) and then January288 (9%). Minimum number of cases was reported in the month of February. The months of May to August and October to January showed slightly elevated number of reported medico-legal cases as shown in Table-II. This study also showed that maximum cases reported in rainy season (July and August) followed by winter (November, December, January) as compared to summer (March, April, May and June).

**Table-II:** Monthly distribution of various Medico Legal Cases.

*Categories*	*Jan*	*Feb*	*Mar*	*Apr*	*May*	*Jun*	*Jul*	*Aug*	*Sep*	*Oct*	*Nov*	*Dec*	*Total*
Blunt injuries	89	70	80	86	76	68	80	104	81	76	86	70	966
RTAs	103	77	91	101	89	93	114	127	89	112	127	107	1230
Sharp injuries	71	47	41	38	51	60	44	51	28	32	55	64	582
Sexual Assault	7	7	6	6	8	9	5	5	1	3	2	2	61
Poisoning	8	12	12	7	6	8	13	14	10	10	23	20	143
Firearm injuries	10	5	13	10	9	6	13	13	11	12	5	14	123

Total	288	218	243	238	239	256	269	314	220	245	298	277	3105

In cases of blunt trauma, sharp weapon injuries and firearm injuries, there was a huge preponderance of victims from rural areas(65%), (62%)and 61% respectively, while urban cases constituting less. The road traffic accidents were high in urban areas (74%) as compared to those reported from rural vicinity (37%). The cases of sexual assault reported more (76%) from urban areas than from rural areasand cases of poisoning reported were almost equal in both urban and rural areas, further details shown in Table-III.

**Table-III:** Various causes of MLC in rural and urban areas (n=3105).

*Type of MLC*	*Male*		*Female*		*Total*

	*Rural N(%)*	*Urban N (%)*	*Rural N (%)*	*Urban N (%)*
Blunt injuries	477(49)	307(32)	148(15)	34(4)	966
Sharp injuries	324(56)	200(34)	35(6)	23(4)	582
Road Traffic Accidents	266(21)	698(57)	47(4)	219(17)	1230
Sexual assault	0(0)	14(23)	15(25)	32(52)	61
Poisoning	34(24)	35(24)	50(35)	24(17)	143
Firearm injuries	47(38)	62(51)	10(8)	4(3)	123

## DISCUSSION

Medicolegal cases represent one of the major groups of all emergencies presented to casualty department of any hospital. The social, demographic and epidemiological transition due to rapid urbanization, mechanization and industrialization has augmented the frequency of such cases.^4^

In the present study a total of 3015 Medico legal cases reported to casualty department of a tertiary care hospital of Rawalpindi. The present study revealed that maximum number of cases were of Road Traffic Accidents (38%) followed by physical assault (32%) and sharp weapon injury (19%). Our findings were consistent with the studyconducted at Nepal.^5^ On the contrary to the study conducted in UK, where prevalence of penetrating trauma was on highest in urban areas with 86.8% and second were firearm injuries with 13.3%.^6^ Use of firearms was found to be less in this study, in contrast to the England & Waleswhere firearms comprise majority of injuries and mortalities.^6^

The findings of the present study showed that road traffic injuries (RTIs) were one of the major causes of MLC cases. RTIs have a yearly incidence of 15 injuries for every 1000 persons andtake lifeof 5000 and hurt 12,000 person in Pakistan every year.^7^ Road traffic accidents may be attributed to nonexistence of safety rules, horrific road infrastructure, avoidance of helmets and seat-belts use, lack of implementation of traffic laws and legislations, careless, rash and negligent driving, and availability of limited trauma care facilities inPakistan.^8^,^9^ Emergency medical services comprising of ambulances and trained paramedics were available only in 5% of the urban areas.^9^

In our study physical trauma accounted for 32% of all cases similar to that reported in India.^10^ In the first national injury survey in Pakistan, the yearly overall incidence of injury was found to be 41 injuries for every 1,000 personscontrary to the low incidence inthe western world.^11^ The pattern of trauma was similar to that reported in some other studies in Pakistan.^11^,^12^ In case of injuries by sharp weapon the age most prone was 20–29 years which was analogous to other studies in Pakistan.^12^ The findings of this study revealed that majority of victims were from below 30 yearsof age (73%), was in concurrence with the generally reported worldwide and a similar age predilection has been reported in other cities of Pakistan.^13^ A male preponderance was also found out by our study showing majority of victims were males as compared to the females similar to other studies.^13^,^14^

This trend of male to female ratio depends on the role of females in a society, in our societies females are less exposed to trauma as compared to males.^13^,^14^ The seasonal variation of accounted cases was probably owing to extremes of weather and poor condition of roads, comparable to other studies in India and Pakistan.^13^,^14^ Our study revealed that majority of victims belonged to urban areasas compared to rural areas, people residing in urban areas were more prone to road traffic accidents, industrial hazards, railway mishaps, fall from high rise buildings and other factors.^14^ This was also revealed that blunt and sharp injuries were reported more from rural areas probably due to instances of quarreling, socio-cultural customs and low literacy rate.^15^ This leads to a huge socioeconomic impact as any kind of trauma can hamper not only the life of the individuals, also their families and societies in general.^15^

From public health perspective, violence especially in its severest form of assault or homicide is a significant predicament, most commonly affecting economically productive age group. The most suitable interventions could be provision of opportunities to engage this age group in various productive measures like sports and recreational activities, enhanced injury related research; improvement in post traumatic care of casualties, road safety education and creating public awareness to prevent these aberrant morbidities in future.

## CONCLUSION

Road traffic injuries are one of the foremost causes of medico legal cases followed by blunt trauma and sharp weapon injuries, comprising a considerable public health problem. Although in the presence of substantial evidence, emerging medico legal cases are neglected epidemic in most of the developingcountries. The magnitude of problem needs to be addressed and comprehensive approach for preventive strategies ought to be developed by public health officials. Further research can be conducted to find out incidence, etiology and outcome of these medicolegal issues for concerned sections of the society.

### Authors’ Contribution

**RM** conceived, designed, data collection & editing of manuscript.

**IA** did data analysis and manuscript writing.

**FR** did review and final approval of manuscript.

**MA** did data collection.

**RM** responsible and accountable for the accuracy or integrity of the work.
